# Effect of natural weed and Siratro cover crop on soil fungal diversity in a banana cropping system in southwestern China

**DOI:** 10.3389/fmicb.2023.1138580

**Published:** 2023-03-22

**Authors:** Yongfen Wang, Wenlong Zhang, Paul H. Goodwin, Si-Jun Zheng, Xundong Li, Shengtao Xu

**Affiliations:** ^1^Institute of Tropical and Subtropical Cash Crops, Yunnan Academy of Agricultural Sciences, Baoshan, Yunnan, China; ^2^Yunnan Key Laboratory of Green Prevention and Control of Agricultural Transboundary Pests, Agricultural Environment and Resources Institute, Yunnan Academy of Agricultural Sciences, Kunming, Yunnan, China; ^3^School of Environmental Sciences, University of Guelph, Guelph, ON, Canada; ^4^Bioversity International, Kunming, Yunnan, China

**Keywords:** cover crop, banana, soil fungal communities, soil microbial diversity, co-occurrence network

## Abstract

**Introduction:**

Natural weed cover and a legume cover crop were examined to determine if they could impact soil fungal diversity as an indicator of soil quality in banana production.

**Methods:**

Banana in Yunnan Province, China, was grown under three treatments: conventional tillage (bare soil), natural weed cover (primarily goosegrass (*Eleusine indica* (L.) Gaerth)), or a cover crop (Siratro (*Macroptilium atropurpureum* (DC.) Urb.)). Analysis of the soil fungal communities between 2017 and 2020 was done by Illumina Miseq high-throughput sequencing.

**Results:**

Most significant effects were in the intercropping area for the treatments, whereas it was rarely observed in the furrow planted with banana. Based on the Shannon and Simpson diversity indices, soil fungal diversity in the intercropping area significantly decreased following planting banana in 2017 with all three treatments. However, both the Shannon and Simpson diversity indices showed that there were significant increases in fungal soil diversity in 2019 and 2020 with natural weed cover or Siratro compared to bare soil. At the end of the experiment, significant increases in fungal genera with Siratro compared to bare soil were observed with *Mortierella*, *Acremonium*, *Plectophaerella*, *Metarhizium* and *Acrocalymma*, and significant decreases were observed with *Fusicolla*, *Myrothecium*, *Exserohilum*, *Micropsalliota* and *Nigrospora*. Siratro resulted in higher stability of the soil fungal microbiome by increasing the modularity and the proportion of negative co-occurrences compared to bare soil. For fungal guilds, Siratro significantly increased saprotrophs_symbiotrophs in 2019 and 2020 and significantly decreased pathogens_saprotrophs in 2020 compared to bare soil.

**Discussion:**

Using Siratro as a cover crop in the intercropping area of banana helped maintain soil fungal diversity, which would be beneficial for soil health with more symbiotrophs and less pathogens in the soil. However, further research is needed to determine the long-term impact of weed or Siratro cover crop on the fungal soil ecosystem and growth of banana.

## 1. Introduction

Banana (*Musa* spp.) is a perennial monocotyledonous plant with the nutrient-rich fruit, which is an important cash crop in tropical and subtropical regions worldwide (Bubici et al., 2019). Conventional banana production in China can result in serious soil degradation, such as soil nutrient loss, acidification, compaction, salinization, and increased disease (Chen et al., 2018) Nevertheless, soil is usually considered an important natural resource for maintaining the function and sustainability of terrestrial ecosystems, which is a living, dynamic and non-renewable resource on a human time scale (Evans et al., 2022). Therefore, sustainability is becoming an urgent issue for agro-ecosystems (Drost et al., 2020), and maintaining beneficial soil properties to improve sustainability is a priority to ensure sustainable banana production in China and worldwide.

Soil physical and chemical properties are traditionally considered key elements of soil fertility, and are considered to be intrinsic and relatively static properties (Taboada et al., 2011). However, soil biological properties are highly dynamic playing crucial roles in soil nutrient cycling and function (Jian et al., 2020). For example, agricultural practices, such as deep tillage and high nitrogen fertilization, can negatively affect soil biology (Nivelle et al., 2016). Intensively managed mono-cropping can result in soil biodiversity losses dramatically reducing key soil functions (Tao et al., 2022). Alternative farming practices provide a way to reduce the negative effects of agricultural production on soil. These include using conservation tillage, rotation, intercrops, fallow periods and cover crops (Serebrennikov et al., 2020).

Cover crops can help retain soil moisture and nutrients, improve soil quality, and enhance the soil productivity (Blanco-Canqui and Ruis, 2020). There have been a number of examples for its use in banana. For example, intercropping banana with a mixture of *Alysicarpus ovalifolius*, *Brachiaria decumbens*, *Chamaecrista rotundifolia*, *Cynodon dactylon*, *Dichondra repens*, *Macroptilium atropurpureum*, *Neonotonia wightii*, *Paspalum notatum*, *Pueraria phaseoloides*, and *Stylosanthes* spp. as a cover crop improved crop productivity and did not result in competition with banana for nitrogen (Tixier et al., 2011). Also, planting a mixture of *Paspalum notatum*, *Neonotonia wightii*, *Pueraria phaseoloides*, and *Stylosanthes guyanensis* as a cover crop increased yield of banana and reduced nematode damage (Djigal et al., 2012). Planting *Canavalia ensiformis*, *Cajanus cajan*, C*rotalaria* spp*., Sorghum bicolor* and *Phaseolus lunatus* as cover crops resulted in significant weed control in banana and increased yield (Ávila et al., 2020).

A common cover crop is Siratro, also known as purple bush-bean [*Macroptilium atropurpureum* (DC.) Urb.] (Chatterjee, 2021). It is a perennial climbing legume with dense vines that originated in tropical and subtropical regions of the Americas (Rojas-Sandoval, 2018). It can improve soil physical–chemical properties, such as soil pH and levels of calcium and magnesium (Espindola et al., 2005), and leaves large amounts of organic residues that can increase organic carbon, nitrogen and potassium levels (Macharia et al., 2011). Such changes can also indirectly affect soil microbial diversity. Examples of its use of a cover crop include planting Siratro to improve soil fertility and maize biomass production (Abayomi et al., 2001), and using it as a green manure for passion-fruit and rice (Werasopon et al., 1998; Gama-Rodrigues et al., 2007). Examples of its use with banana include using it as a green manure to increase nutrients, particularly N, Ca and Mg (Espindola et al., 2006), and rotating it with banana to reduce plant-parasitic nematode damage (Risède et al., 2009).

Soil microbial diversity is considered to be a key parameter in evaluating soil health in agricultural ecosystems (Tahat et al., 2020). Microbial functional diversity as well depends on the diversity of the microbes present (van Capelle et al., 2012). However, the biogeochemical processes affected by soil microorganisms are also highly sensitive to variation in environmental factors (Cycoń et al., 2019). One way to increase soil microbial diversity is to increase plant diversity (Venter et al., 2016). This can be done with cover crops. For example, a cover crop of black medic resulted in higher alpha diversity in soil bacterial communities (Lupwayi et al., 2018), a cover crop mixture of triticale, rye and common vetch resulted in higher soil fungal diversity (Schmidt et al., 2019). Increasing soil fungal diversity is important as soil fungi can benefit plants, such as by increasing phosphate availability (Barroso and Nahas, 2005), decaying organic matter to release nutrients (Argiroff et al., 2022), degrading toxic compounds in soil (Chang et al., 2016), increasing antagonisms to pathogenic microbes (Miao et al., 2016) and improving soil structure by binding soil particles (Miller and Jastrow, 2000). Despite the benefits reported for cover crops in improving soil health and plant production, there is little work done on cover crops for such benefits in banana cropping systems.

In this study, a banana plantation was established in 2017 in a dry hot valley in southwestern China with the rows between the banana plants having either conventional tillage to maintain bare soil, a natural weed cover allowed to develop, or Siratro planted as a cover crop. The soil beneath the banana plants and between the plants was analyzed for fungal diversity, functional guilds and co-occurrence until 2020. The goal was to better understand how soil fungi respond to the cover crop to improve banana plant soil management.

## 2. Materials and methods

### 2.1. Site description

A site was chosen at the Science and Technology Demonstration Park of Institute of Tropical and Subtropical Cash Crops of Yunnan Academy of Agricultural Sciences, Lujiangba (N24°57′58′，E 98°53′14′) Baoshan, Yunnan, China. The climate type is dry-hot subtropical. Mean precipitation was approx. 750 mm, mean annual pan evaporation was greater than 2,100 mm, mean annual temperature was 21.3°C, maximum temperature was 40.4°C, minimum temperature was 0.2°C, annual accumulated temperature ≥ 10°C was 7,800°C, annual sunshine duration was 2,333.7 h, altitude was 700 m, and relative humidity was 70%. The soil was sandy loam soil. The soil physio-chemical parameters were pH 6.71, organic matter 11.36 g kg^−1^, alkaline nitrogen 57.84 mg kg^−1^, available phosphorus 28.50 mg kg^−1^ and available potassium 95.86 mg kg^−1^.

### 2.2. Plot design, treatments, and soil sampling

**Figure 1:**
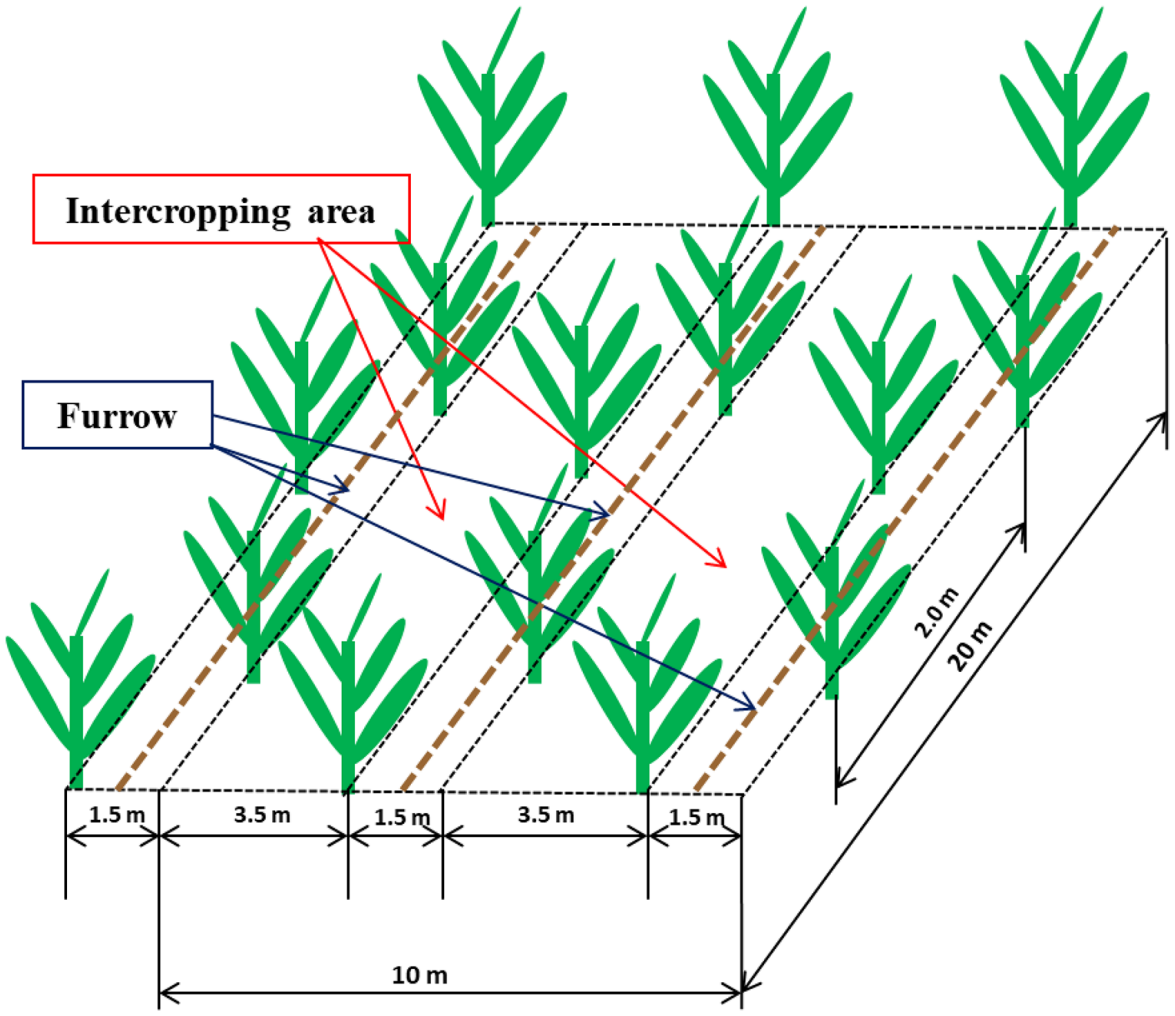
Diagram showing the 3.5 m intercropping area and 1.5 m furrow containing the banana plants within an individual plot.

A randomized complete block factorial design was established in 2017 with four replications with each plot was 135 m^2^ with 35 banana plants (cv. Yunjiao No. 1). The treatments were conventional tillage with bare soil (CT), natural weed cover that was mainly goosegrass, *Eleusine indica* (WC) and Siratro cover (SC). Each plot contained 3.5 m and 1.5 m rows defined as the intercropping area and furrow, respectively (Figure 1). Banana seedlings were planted 26 July 2017 in the furrows, and the CT, WC and SC treatments were applied to the intercropping area. CT treatment was sprayed with herbicide monthly to remove weeds, WC treatment allowed weeds to grow into the intercropping area, except for 30 cm from the banana seedlings in year one to avoid shading of the banana plants, and SC treatment was *M. atropurpureum* planted in 2017 at a density of 22.5 kg hm^−2^ to provide more than 60% plant coverage of the intercropping area. A micro-sprinkler irrigation system was used to apply water in the furrows once a week in the dry season (October to May) as well as several times as needed in rainy transition season (August and September). No irrigation was done in the rainy season (June to July), and there was no irrigation of the intercropping area. Fertilizer (40% urea, 40% compound (N: P: K = 15: 15: 15) and 20% potash) was applied twice a month at 1.5 kg per plant per year, and there was no fertilization of the intercropping area.

To collect soil for DNA analysis, soil was sampled from five random locations in each plot using a manual soil auger to the depth of the tilled layer (30 cm) in July, which was the time of planting banana in 2017 and the period of fastest growth of the cover crop during the warm late rainy season each year until 2020. Soil from the five locations per plot were combined to form one sample per plot. The soil was sieved (≤2 mm), and approximately 20 g of soil was stored at −80°C for DNA extraction.

### 2.3. DNA extraction and sequencing

Total genomic DNA was extracted from each sample using the OMEGA Soil DNA Kit (D5625-01) (Omega Bio-Tek, Norcross, GA, United States), following the manufacturer’s instructions, and stored at-20°C. The quantity and quality of the DNAs were measured using a NanoDrop ND-1000 spectrophotometer (Thermo Fisher Scientific, Waltham, MA, United States) and agarose gel electrophoresis. The concentration of DNA, OD 260/280 ratio, OD260/230 and the number of analyzed sequences of each sample are presented in Supplementary Table S1, and the number of total NGS reads, quality filtered reads, read length and SE/PE reads per sample are given in Supplementary Table S2.

PCR amplification of the fungal ITS1 region was performed using the forward primer ITS5F (5′- GGAAGTAAAAGTCGTAACAAGG-3′) and the reverse primer ITS1R (5′- GCTGCGTTCTTCATCGATGC-3′). Sample-specific 7-bp barcodes were incorporated into the primers for multiplex sequencing. The PCRs contained 5 μL Q5 reaction buffer (5×), 5 μL Q5 HighFidelity GC buffer (5×), 0.25 μL Q5 High-Fidelity DNA Polymerase (5 U/μL), (2 μL 2.5 mM) dNTPs, 1 μL each 10 μM ITS5F and ITS1R primers, 2 μL DNA template, and 8.75 μL ddH_2_O. Thermal cycling consisted of initial cycle of 98°C for 2 min, followed by 25 cycles of 98°C for 15 s, 55°C for 30 s, and 72°C for 30 s, with a final cycle of 5 min at 72°C. PCR amplicons were purified with Agencourt AMPure Beads (Beckman Coulter, Indianapolis, IN, United States) and quantified using the PicoGreen dsDNA Assay Kit (Invitrogen, Carlsbad, CA, United States). Paired-end 300 bp sequencing was performed using the Illlumina MiSeq platform with MiSeq Reagent Kit v3 (Shanghai Personal Biotechnology Co., Shanghai, China). The sequence data for all samples were deposited at NCBI, accession number PRJNA894310.

### 2.4. Sequence analysis

Microbiome analysis was performed with QIIME2^1^ with slight modifications. Briefly, raw sequence data were demultiplexed using the demux plugin followed by primer removal with the cutadapt plugin (Magoč and Salzberg, 2011). Sequences were then quality filtered, denoised, merged and chimeras removed using the DADA2 plugin (Callahan et al., 2016). Non-singleton amplicon sequence variants (ASVs) were aligned with mafft (Katoh et al., 2002), and trees were constructed with fasttree2 (Price et al., 2009). Taxonomy was assigned to ASVs using the classify-sklearn naïve Bayes taxonomy classifier in the feature-classifier plugin (Bokulich et al., 2018) against the UNITE Release 8.0 Database.

### 2.5. Statistical analysis

All analysis was done with R 4.1.2.^2^ Alpha diversity indices were calculated by analyzing Chao1 richness and Shannon diversity in the Vegan package in R (Oksanen et al., 2013). β-diversity and construction of non-metric multidimensional scaling (NMDS) plots were also performed in the Vegan package in R. Normalized ASVs were analyzed using Bray Curtis metrics (Bray and Curtis, 1957). Bray-Curtis distance matrices were subjected to multivariate analysis of variance (PERMANOVA) (Anderson, 2001) to compare fungal community composition and abundance using the Adonis function with a permutation number of 999 in the Vegan package in R. Linear discriminant analysis (LDA) effect size (LEfSe) was used to compare taxonomic features between groups by the Python LEfSe package in R with an LDA threshold of 2.0 and an alpha value of 0.05. A heatmap was constructed for the statistically dominant genus (LDA *p* < 0.05) using the ‘heatmaply’ package in R (Galili et al., 2018). Fungal ASVs were assigned into functional guilds using the online application FUNGuild^3^ (Nguyen et al., 2016). Co-occurrence patterns were reconstructed by Hmisc package in R and Gephi 0.9.2, A co-occurrence was considered to be robust if the Spearman’s correlation coefficient (*r*) was >0.80 and *p* < 0.05. Network stability was measured by the proportion of negative or positive correlations and the modularity (Gao et al., 2021).

## 3. Results

### 3.1. Soil fungal diversity and richness

**Figure 2:**
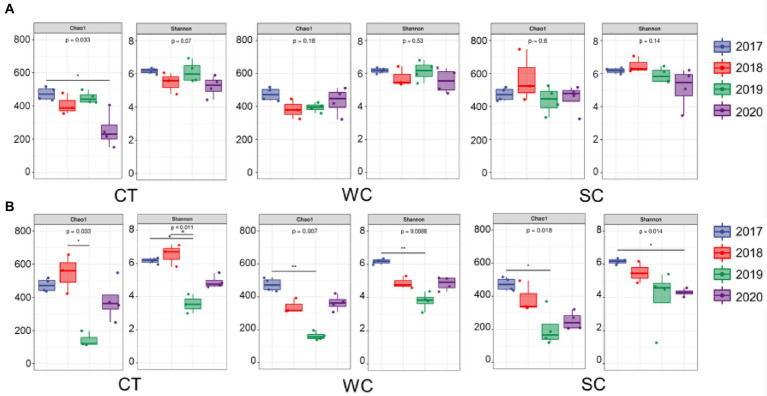
Chao1 and Shannon soil fungal diversity indices over years with conventional tillage (CT), weed cover (WC), and Siratro cover (SC) treatments in the intercropping area **(A)**, and furrow **(B)**. *, **, *** indicates *p* ≤ 0.05, 0.01, and 0.001, respectively.

For the intercropping area with CT treatment, the Chao1 diversity index of soil fungi showed significantly less diversity after planting in 2020 compared to before planting in 2017, and the Shannon diversity index showed lower diversity in 2018 and 2020 compared to 2017 (Figure 2A). With WC treatment, there was significantly less diversity compared to 2017 in 2018 and 2019 with the Chao1 index and 2018 with the Shannon index. With SC treatment, there was only a significant difference in diversity between 2017 and 2020 with the Shannon index. Thus, soil fungal diversity in the intercropping area was generally least reduced with the SC treatment and most reduced with the CT treatment. A comparison between years showed that no treatment resulted in significant differences in soil fungal diversity in the intercropping areas (Supplementary Figure S1).

In the furrow area of the CT plots, there was a significant decrease in diversity between pre-planting in 2017 and post planting in 2019 and 2020 detected with both indices (Figure 2B). In the furrow area of the WC and SC plots, both indices showed that diversity significantly decreased detected in all years after planting compared to pre-planting Thus, soil fungal diversity was always detected to be reduced after planting, except for 2018 in the furrow with CT treatment. It appears that the treatments were less effective in maintaining fungal soil diversity in the furrow where the banana plants were located than in the intercropping area. Similar to the intercropping area, no treatment resulted in significant differences in soil fungal diversity between years in the furrow area (Supplementary Figure S1).

### 3.2. Soil fungal community composition

**Figure 3:**
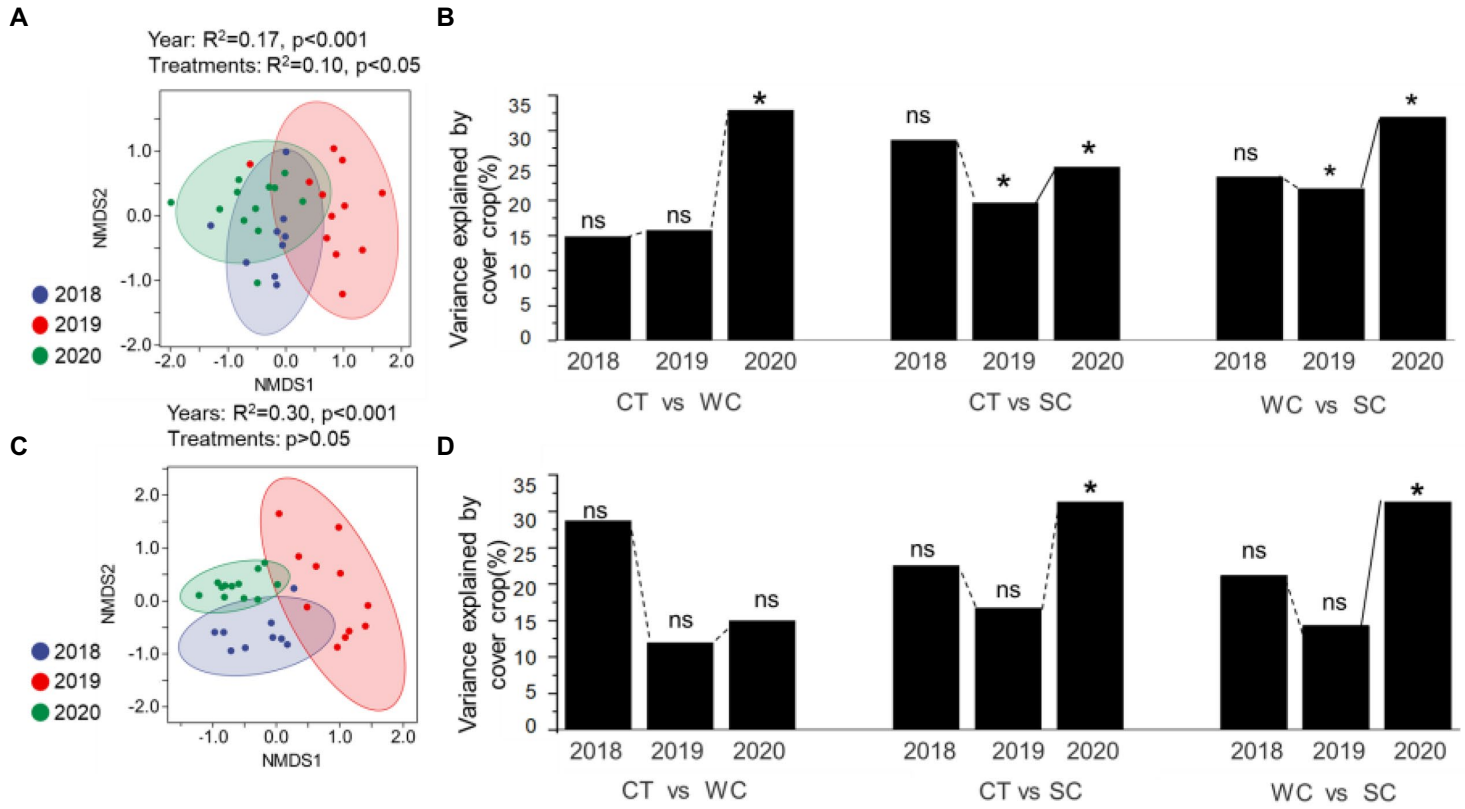
Soil fungal community composition variation over years with conventional tillage (CT), weed cover (WC), and Siratro cover (SC) treatments. NMDS and PERMANOVA analysis of the intercropping area (**A**,**B**, respectively), and furrow (**C**,**D**, respectively). *Indicates significance at *p* ≤ 0.05, and ns indicates not significant.

Analysis of the soil fungal community composition by NMDS (Figure 3A) and PERMANOVA (Figure 3B) showed that it was significantly affected in the intercropping area by year primarily in 2019 and 2020 for all treatment comparisons. In the furrow area, soil fungal community composition by NMDS (Figure 3C) and PERMANOVA (Figure 3D) was significantly affected only in 2020 for the CT-SC and WC-SC treatment comparisons. Figure 3A shows clustering but the separation of the clusters is much less than the intercropping area in Figure 3C. While we mostly focused on the intercropping area with the treatments, but the furrows were somewhat affected by the same management. While PERMANOVA analysis showed significant effects of the treatments on soil fungal community in both the intercropping area and furrow. These results indicate that both areas were affected by year and treatment, but more significant differences were observed by treatment for the intercropping area, which is where the treatments were applied. Consistent with this is that the SC treatment thus appeared to have earlier effects on soil fungal community composition in the intercropping area than the furrow (Supplementary Figure S2).

### 3.3. Dominant soil fungal groups

**Figure 4:**
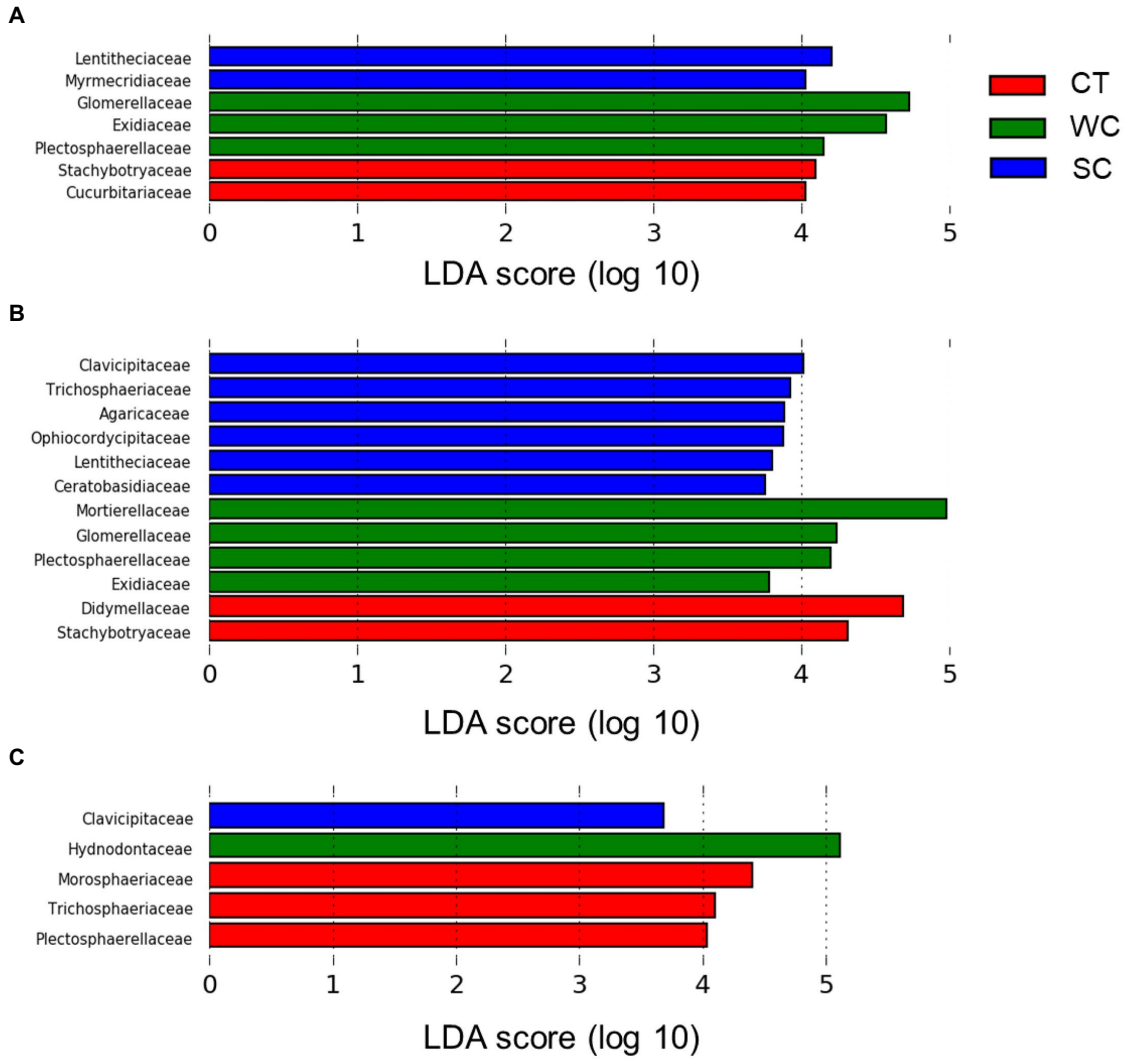
LDA score distribution at the fungal family level for conventional tillage (CT), weed cover (WC), and Siratro cover (SC) treatments in the intercropping area in 2019 **(A)**, 2020 **(B)** and the furrow in 2020 **(C)**.

At the fungal family level, there were significant differences in relative abundance between treatments using Lefse analysis in the intercropping area in 2019 and 2020 (Figures 4A,B). The number of families with the highest relative abundance remained at two in 2019 and 2020 with CT treatment, whereas it increased from three to four with the WC treatment and two to five with the SC treatment. The change over time in the number of highest abundant fungal families was mostly but not entirely due to the new families being included. There were also significant differences in relative abundance between treatments in the furrow in 2020 (Figure 4C). Unlike the intercropping area, however, most of the families with highest relative abundance in 2020 were with the CT treatment in the furrow. The only shared fungal family in this analysis between the intercropping area and furrow in 2020 was the Clavicipitaceae with the SC treatment.

At the end of the experiment in 2020, significant differences between treatments were observed among the 30 most abundant genera in the intercropping area (Supplementary Table S3). For SC compared to CT treatment, there were significantly higher levels of *Mortierella, Acremonium, Plectophaerella, Metarhizium* and *Acrocalymma*, but significantly lower levels of *Fusicolla, Myrothecium, Exserohilum, Micropsalliota* and *Nigrospora*. For SC compared to WC treatment, significantly higher levels of *Mortierella, Acremonium, Plectosphaerella* and *Acrocalymma*, but significantly lower levels of *Fusicolla, Myrothecium, Micropsalliota, Nigrospora, Pyrenochaetopsis, Corynascella, Poaceascoma*, and *Dokmaia*, were observed. For WC compared to the CT treatment, there were significantly higher levels of *Fusicolla, Metarhizium, Micropsalliota, Nigrospora* and *Dokmaia*, but significantly lower levels of *Trechispora* and *Mycosphaerella*.

At the end of the experiment in 2020, significant differences were also observed between treatments among the 30 most abundant genera in the furrow (Supplementary Table S4). There were significantly higher levels of *Trechispora* and significantly lower levels of *Acrocalymma, Micropsalliota, Fusicolla, Nigrospora* and *Metarhizium* with SC compared to CT treatment, and there were significantly higher levels of *Trechispora* and significantly lower levels of *Fusicolla* and *Metarhizium* with SC compared to WC treatment. For WC compared to CT treatment, significantly higher levels of *Micropsalliota* and *Fusicolla* were observed, but no genera significantly were observed with lower levels.

### 3.4. FUNguild functional prediction

**Figure 5:**
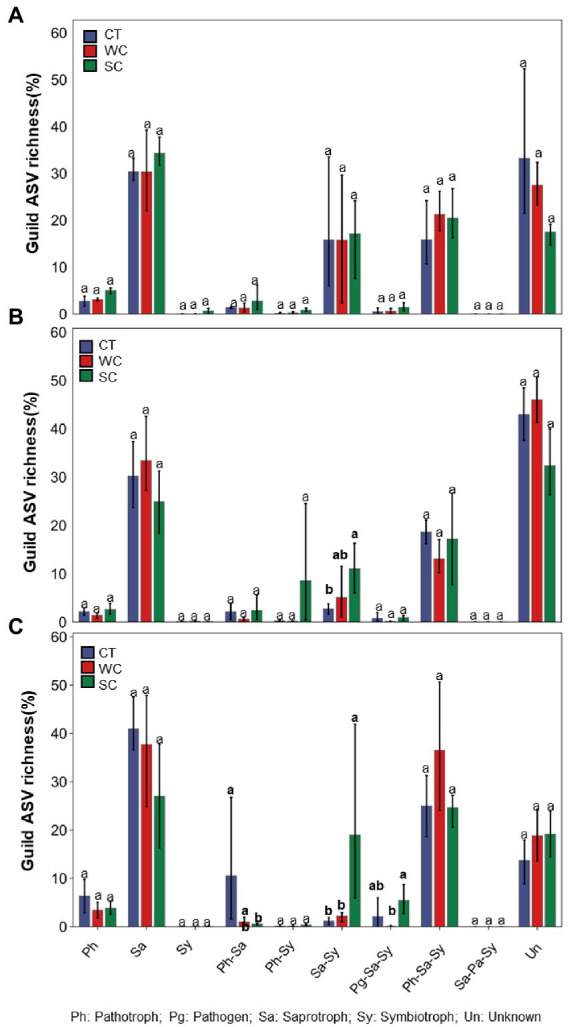
Funguild predictions of functional fungal guild ASV richness of the conventional tillage (CT), weed cover (WC) and Siratro cover (SC) treatments in the intercropping area in 2018 **(A)**, 2019 **(B)** and 2020 **(C)**. Means within the same year followed by the same letters are not significantly different at *p* = 0.05 according to a protected LSD test.

Significant differences in functional prediction using FUNguild were observed due to treatments in intercropping area in 2018, 2019, and 2020 (Figures 5A–C). Compared to CT treatment, SC treatment resulted in significantly higher saprotroph_symbiotroph guild in 2019 and 2020, and significantly lower pathotroph_saprotroph guild in 2020. There was also significantly higher pathogen_saprotroph_symbiotroph guild with SC compared to WC treatment in 2020. The only significant difference between treatments in the furrow was for a significantly lower unknown guild with SC treatment in 2018, 2019, and 2020 (Supplementary Figures S3A–C).

### 3.5. Co-occurrence network analysis

**Figure 6:**
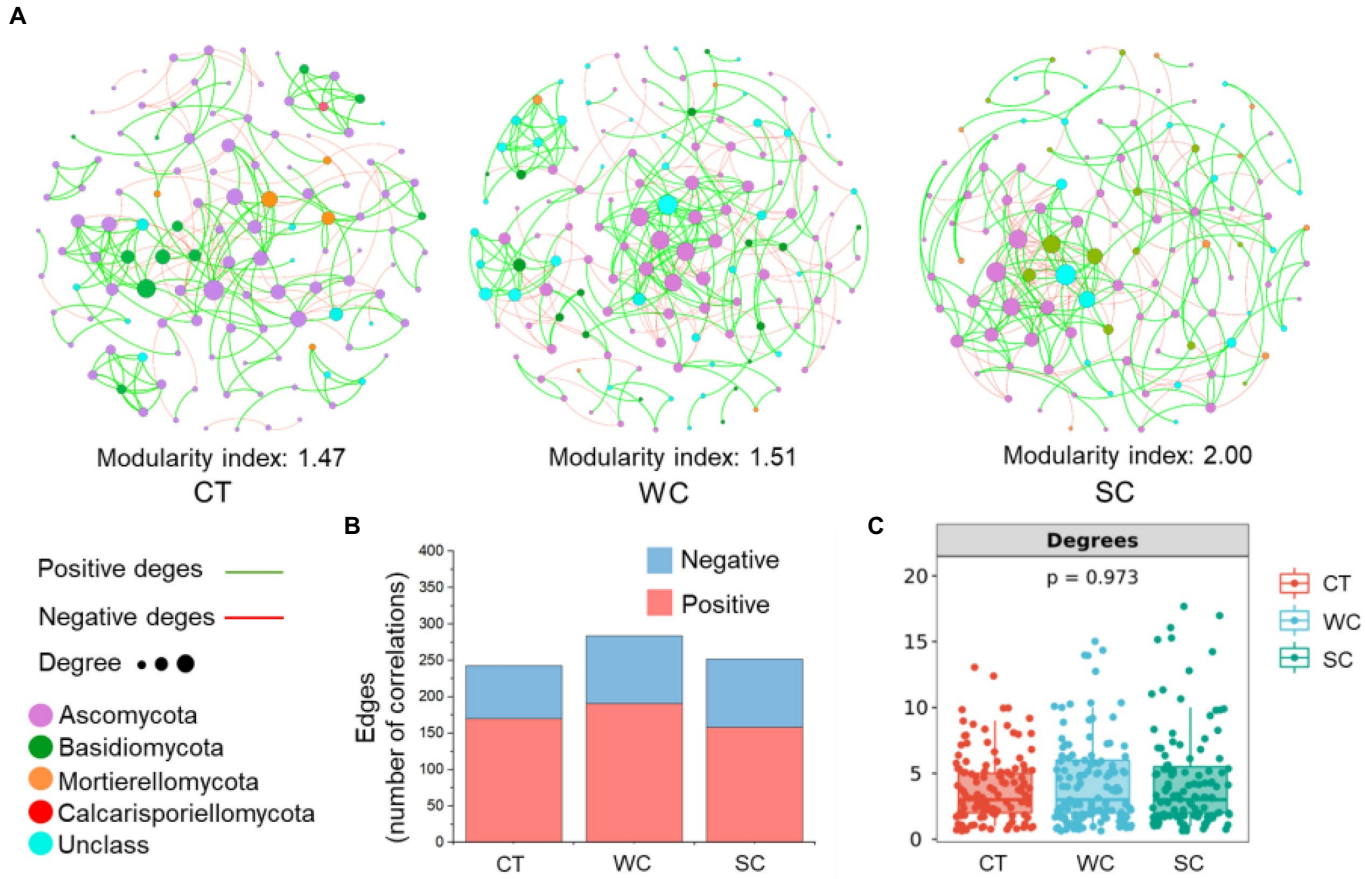
Co-occurrence networks with the conventional tillage (CT), weed cover (WC) and Siratro cover (SC) treatments in intercropping area. Modularity index and co-occurrence networks at the fungal phylum level in 2019 and 2020 **(A)**. Number of positive and negative edges of two co-occurrences **(B)**. Degrees of co-occurrences **(C)**.

PERMANOVA analysis of soil fungal community members with a relative abundance above 0.1% revealed significant differences between treatments in the intercropping area in 2019 and 2020, showing that the data from those years were suitable for the co-occurrence network analysis (Supplementary Table S3). SC treatment resulted in a higher stability of the soil fungal community with a modularity index of 2.00 compared to 1.47 and 1.51 for CT or WC treatments, respectively (Figure 6A). Based on edge analysis, the number of positive co-occurrences was 170, 191, and 158 for CT, WC and SC treatments, respectively, whereas the number of negative co-occurrences was 73, 93, and 94 for CT, WC, and SC treatments, respectively (Figure 6B). Thus, WC treatment had the most positive co-occurrences, and SC treatment had the most negative co-occurrences among the treatments. There were no significant differences in the degrees of the soil fungal community among the treatments indicating no significant differences in soil fungal community complexity (Figure 6C). PERMANOVA analysis of soil fungal community with a relative abundance more than 0.1% revealed no significant differences between treatments in 2018, 2019, and 2020 (Supplementary Table S5). Thus, that data in the furrow area was not suitable for the co-occurrence network analysis.

## 4. Discussion

Perennial cover crops are often used to improve soil properties of tropical fruit crops, even without the need to plow and incorporate the cover crop into the soil (Wei et al., 2021; Freidenreich et al., 2022). However, they are more difficult to use in banana cropping systems compared to other tropical fruits, as banana leaves create a large amount of shade limiting the growth of most cover crops. In addition, many banana producers, such as those in Yunnan province in China, do not use cover crops as they believe that cover crops would compete with banana for water and fertilizer. However, the lack of cover crops has contributed to banana soil degradation and possibly to higher levels of root diseases, such as banana Fusarium wilt disease (Shen et al., 2018; Hong et al., 2020). One benefit of cover crops is to increase soil fungal diversity and stability with crops, such as an alfalfa cover crop in apple orchards (Wang et al., 2022) and barley or vetch cover crops in sunflower or maize fields (García-González et al., 2016). Soil fungi are sensitive to the environmental factors, such as soil moisture, pH, temperature and organic matter, all of which can be affected by cover crops (Hamman et al., 2007; Wang et al., 2014).

While Siartro has been widely studied as a cover crop with a wide variety of crops worldwide including banana (Werasopon et al., 1998; Abayomi et al., 2001; Espindola et al., 2006; Gama-Rodrigues et al., 2007; Risède et al., 2009), it has been studied in China much less often, but with reports showing benefits in orange and mango orchards (Li et al., 1996; Dong et al., 2016). As this study was too short to determine effects on banana yield, the impact of the Siartro cover crop was assessed by examining the soil fungal microbiome over 3 years comparing it to conventional tillage where there was bare soil, which is the most common current practice in Yunnan province, or allowing weeds to naturally invade, primarily goosegrass, which is also often observed in banana plantations in that region.

In the intercropping area, the Siratro cover crop treatment resulted in a generally much less reduced soil fungal diversity and richness after planting banana compared to conventional tillage or a natural weed cover. In the furrow, however, soil fungal diversity and richness showed a decreasing trend with all treatments, including Siratro, compared to soil before banana was planted. Soil fungal community composition was also more rapidly affected by treatments in the intercropping area than in the furrow. The greater effect of Siratro cover crop where it was grown in the intercropping area compared to the adjacent furrow could be due to the localized nature of the rhizosphere effect where nutrients and other compounds are released in root exudates affecting microbes in the adjacent soil (Dotaniya and Meena, 2015). The rhizosphere likely was the dominant effect as the Siratro cover crop in this study was perennial and not incorporated into the soil resulting in plant residues being limited to senescent tissues. Legume root exudates contain nutrients, such as organic acids, flavonoids, and fatty acids, and signaling molecules, such as flavonoids and strigolactones, that affect soil fungi (Sugiyama and Yazaki, 2012). In contrast, the furrow had no Siratro planted and had virtually no naturally occurring weeds due to shading by the banana leaves, and thus soil fungi would be affected only by the rhizosphere effect from the banana roots.

Among two different cover plants in this study, Siratro showed a greater effect in the intercropping area than the naturally occurring goosegrass. Goosegrass is a highly invasive weed that creates significant competition to crops, negatively affecting crop yields, such as in cotton and maize (Rambakudzibga et al., 2002; Wu et al., 2015). It can also increase diseases by acting as an alternative host for pathogens, such as for the Fusarium wilt fungus of banana (Catambacan and Cumagun, 2022). However, goosegrass will still affect the soil microbiome through its rhizosphere effect, which can have beneficial impacts on soil microbes, such as increasing bacterial populations for bioremediation (Lu et al., 2010). Goosegrass belongs to the Poaceae and is an aggressively reseeding annual, while Siratro belongs to the Fabaceae and is a perennial. In addition to belonging to different plant families with different root exudate compositions and interactions with soil microbes, Siratro would thus be affecting the soil microbiome throughout the year, while goosegrass would die in the winter time, thus affecting the soil microbiome less *via* root exudates compared to Siratro and more *via* tissue decay. Goosegrass is not used as a cover crop, likely because of its aggressiveness, whereas Siratro has often been shown to be a desirable cover crop providing benefits to soil quality (Kocira et al., 2020), soil nitrogen (Virk et al., 2022) and soil microbial populations (Cattelan and Vidor, 1990).

Among the 30 most abundant fungal genera in the intercropping area, 23 would be considered saprotrophs, whereas *Acremonium* and *Trichoderma* would be considered symbiotrophs, *Metarhizium* would be considered an insect pathotroph, and *Exserohilum*, *Aspergillus*, *Plectosphaerella* and *Mycosphaerella* would be considered plant pathotrophs. The Siratro cover crop resulted in higher saprotroph_symbiotroph and lower pathotroph_saprotroph guilds compared to conventional tillage. In contrast, goosegrass weed cover resulted in only lower fungi in the unknown guild compared to conventional tillage. Among 30 most abundant fungal genera in the intercropping area, 5 genera were significantly increased with Siratro cover crop compared to conventional tillage, including *Acremonium* that could be contributing to the increase in saprotrophs_symbiotrophs. Also among those, 5 genera were significantly decreased with Siratro relative to conventional tillage, and *Mortierella, Acrocalymma*, *Myrothecium* and *Exserohilum* could be contributing to the decrease in pathotrophs_saprotrophs. However, less abundant fungal genera would also have an impact. Some other examples where cover crops affected the abundance of soil fungal guilds are Wei et al. (2021) where grass cover crops increased pathotrophs and decreased saprotrophs, Benitez et al. (2016) where legume cover crops increased symbiotrophs, and Aiyer et al. (2022) where a sorghum–sudangrass cover crop decreased pathotrophs and increased symbiotrophs while an alfalfa cover crop increased pathotrophs. Thus, the effect of cover crops on soil fungal guilds appears to highly dependent upon the type of plant used. However, the use of the FUNGuild database in this and other studies has limitations as the existing literature on soil fungi is also limited with the functions of approx. 60% of soil fungi yet to be determined (Nguyen et al., 2016).

Among the 30 most abundant genera in the intercropping area, the Siratro cover crop significantly increased *Mortierella, Acremonium, Plectophaerella, Metarhizium* and *Acrocalymma* numbers relative to conventional tillage, which could impact banana production. Increased levels of *Mortierella* spp. could benefit banana, such as *Mortierella capitata* that increased available phosphorus and mycorrhizal populations with maize (Li et al., 2020) and *Mortierella elongata* that increased *Populus* biomass (Zhang et al., 2020). Increased levels of *Acremonium* spp. could benefit banana as many species can enhance plant insect resistance (Breen, 1994) and plant stress resistance, such as drought (Siegel, 1993). However, increased levels of *Plectophaerella* spp. might be harmful, such as *Plectosphaerella cucumeria* that causes root rot of banana (Kanakala and Singh, 2013). Increased levels of *Metarhizium* spp. might benefit banana as three entomopathogenic *Metarhizium* spp. increased maize yields by colonizing roots allowing seedlings to establish earlier (Liao et al., 2014). Increased levels of *Acrocalymma* spp. might also benefit banana as *Acrocalymma vagum* promoted growth of liquorice plants (He et al., 2019).

Compared to conventional tillage, the Siratro cover crop significantly decreased *Fusicolla, Myrothecium, Exserohilum, Micropsalliota* and *Nigrospora* in the 30 most abundant genera in the intercropping area. Decreased levels of *Fusicolla* spp. could be unfavorable for banana as *Fusicolla violacea* was shown to have suppressive activity against many fungal phytopathogens and a biocontrol agent of soft rot of kiwifruit (Li et al., 2021). Decreased levels of *Myrothecium* spp. could benefical as various *Myrothecium* spp. are broad host range foliar plant pathogens (Quezado Duval et al., 2010) Decreased levels of *Exserohilum* spp., could also be favorable for banana as *Exserohilum rostratum* causes banana leaf spot in China (Lin et al., 2011). However decreased levels of *Nigrospora* spp. might not be beneficial as *Nigrospora* spp. are important for leaf decay fungi acting as primary colonists of fallen banana leaves (Meredith, 1962). Decreased levels of *Micropsalliota* spp. could also be negative as is it a common a saprotrophic basidiomycete (Hussain et al., 2022), which could be involved in the decomposition of banana crop residues. However, potential positive or negative effects on banana are not conclusive until analysis can be done at the fungal species level.

Based on the co-occurrence network analysis, the Siratro cover crop resulted in a higher stability of the soil fungal community compared to the other treatments. It was also notable for the lowest number of positive co-occurrences among the treatments but had a similar number of negative co-occurrences as that with goosegrass, which was higher than with conventional tillage. Higher stability of the soil fungi could be considered a positive effect for banana production as ecosystem stability and fungal plant pathogens are negatively correlated (Liu et al., 2022). A lower number of positive co-occurrences indicates reduced cooperative relationships, and a higher number of negative co-occurrences indicates increased levels of competition and antagonism (Coyte et al., 2015). One possibility is that this is a reflection of the rhizosphere effect with a cover crop compared to no rhizosphere effect with conventional tillage. However, there were no significant differences in soil fungal community complexity between treatments, which indicates that changes in soil fungi due to Siratro, goosegrass or conventional tillage reflected shifts in the soil fungal community rather than adding new fungal genera to it.

A major problem with cover crops in banana plantations is that banana leaves can greatly inhibit the cover crop due to shading. In this study, an intercropping area was created, which would be considered wider than the standard cropping method for commercial bananas. This permitted the Siartro cover crop to have less shading from the banana, at least in the first few years of growth. While the intercropping area is wider than in typical commercial production, it still resulted in sufficient banana planting density. Another benefit of a relatively wide intercropping area was that it allowed for mechanized tillage in the intercropping area. Soil fungal diversity showed that changes differed over the years of the study depending upon whether Siratro, goosegrass or bare soil was present in the intercropping area. Further work is needed to determine which of those changes might be beneficial such as by decreasing pathogens and increasing symbionts, harmful such as by decreasing plant debris decay, or have no significant impact on banana production. However, there were no significant differences in soil fungal diversity in the intercropping area between the treatments in any particular year. Schmidt et al. (2019) reported that a mixture of cover crops including triticale, rye and common vetch significantly increased fungal community diversity in tomato-cotton rotations over 14 years. Also, soil fungal diversity was significantly increased with an alfalfa cover crop over 5 years in apple orchards (Wang et al., 2022). Thus, it may take more years to observe changes in soil fungal diversity than was used in this study. As well, it will take more years to observe if the treatments altered banana yield and quality. Despite this, this study shows that the combination of wide intercropping area with a Siartro cover crop does have an impact on soil fungi and shows potential to improve banana production.

## 5. Conclusion

In this study, a Siratro cover crop in the intercropping area of banana helped maintain the diversity of the soil fungal community, unlike bare soil. This correlated with higher saprotrophs-symbiotrophs and lower pathotrophs-saprotrophs compared to bare soil. This could benefit banana growth by increasing the potential for beneficial fungi forming mutualistic interactions with roots and reducing the potential for plant pathogenic fungi to damage roots. This would be beneficial for banana soil health. Based on this study, it appears that intercropping with Siratro could be a simple technique to maintain a soil supporting sustainable banana production. However, future work is needed to extend the study to understand the impacts on banana yield and quality as well as the occurrence of root diseases.

## Data availability statement

The datasets presented in this study can be found in online repositories. The names of the repository/repositories and accession number(s) can be found in the article/Supplementary material.

## Author contributions

YW conceived, performed the experiment, analyzed the data, and wrote the manuscript. WZ conceived and analyzed the data. PG revised the manuscript. S-JZ revised the manuscript. XL conceived, designed the experiment, and revised the manuscript. SX conceived, designed, performed the experiment, wrote, and prepared the manuscript. XL and SX supervised the research and provided funding support. All authors contributed to the article and approved the submitted version.

## Conflict of interest

The authors declare that the research was conducted in the absence of any commercial or financial relationships that could be construed as a potential conflict of interest.

## Publisher’s note

All claims expressed in this article are solely those of the authors and do not necessarily represent those of their affiliated organizations, or those of the publisher, the editors and the reviewers. Any product that may be evaluated in this article, or claim that may be made by its manufacturer, is not guaranteed or endorsed by the publisher.

## Footnotes

^1^ https://docs.qiime2.org/2019.4

^2^ https://cran.microsoft.com/snapshot/2022-02-11/bin/windows/base/

^3^ http://www.stbates.org/guilds/app.php
