# Supplementary material for: Effect of natural weed and Siratro cover crop on soil fungal diversity in a banana cropping system in southwestern China
Source: Front Microbiol. 2023 Mar 22;14:1138580. doi: 10.3389/fmicb.2023.1138580 (PMC10073746; doi:10.3389/fmicb.2023.1138580)
Supplement: Supplementary file 1 [file Data_Sheet_1.docx]

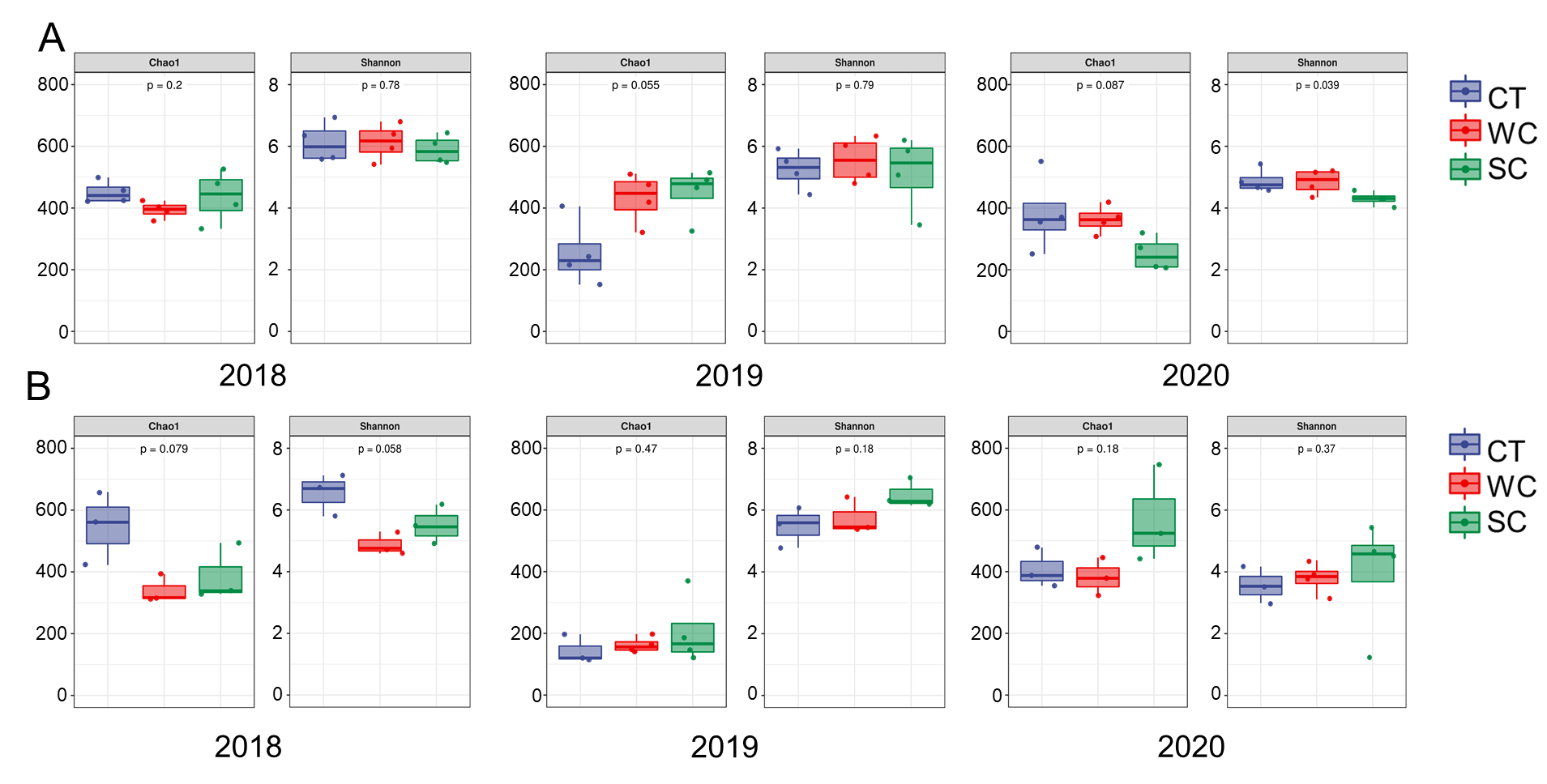


Fig. S1 Chao1 and Shannon soil fungal diversity indices the conventional tillage (CT), weed cover (WC) and Siratro cover (SC) treatments in 2018, 2019 and 2020. *, **, *** is for P ≤ 0.05, 0.01 and 0.001, respectively.


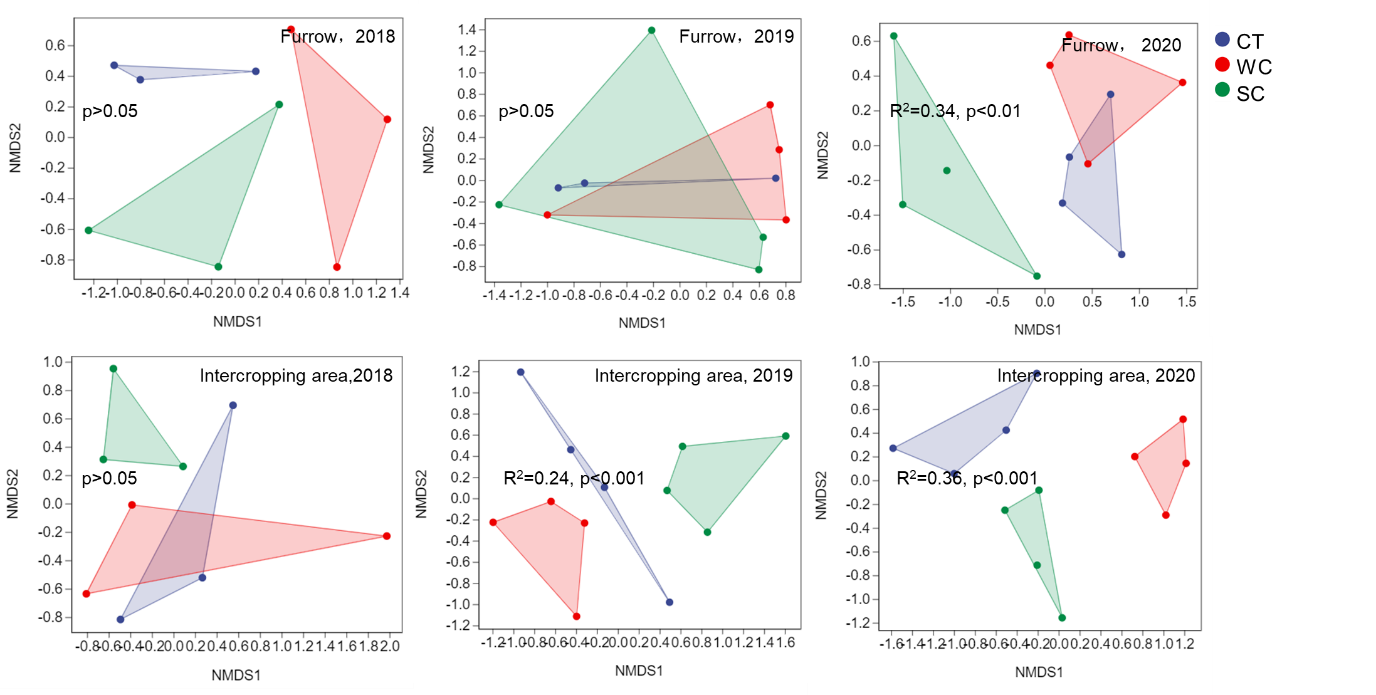


Fig. S2 Soil fungal community composition in 2018, 2019 and 2020 with conventional tillage (CT), weed cover (WC) and Siratro cover (SC) treatments.


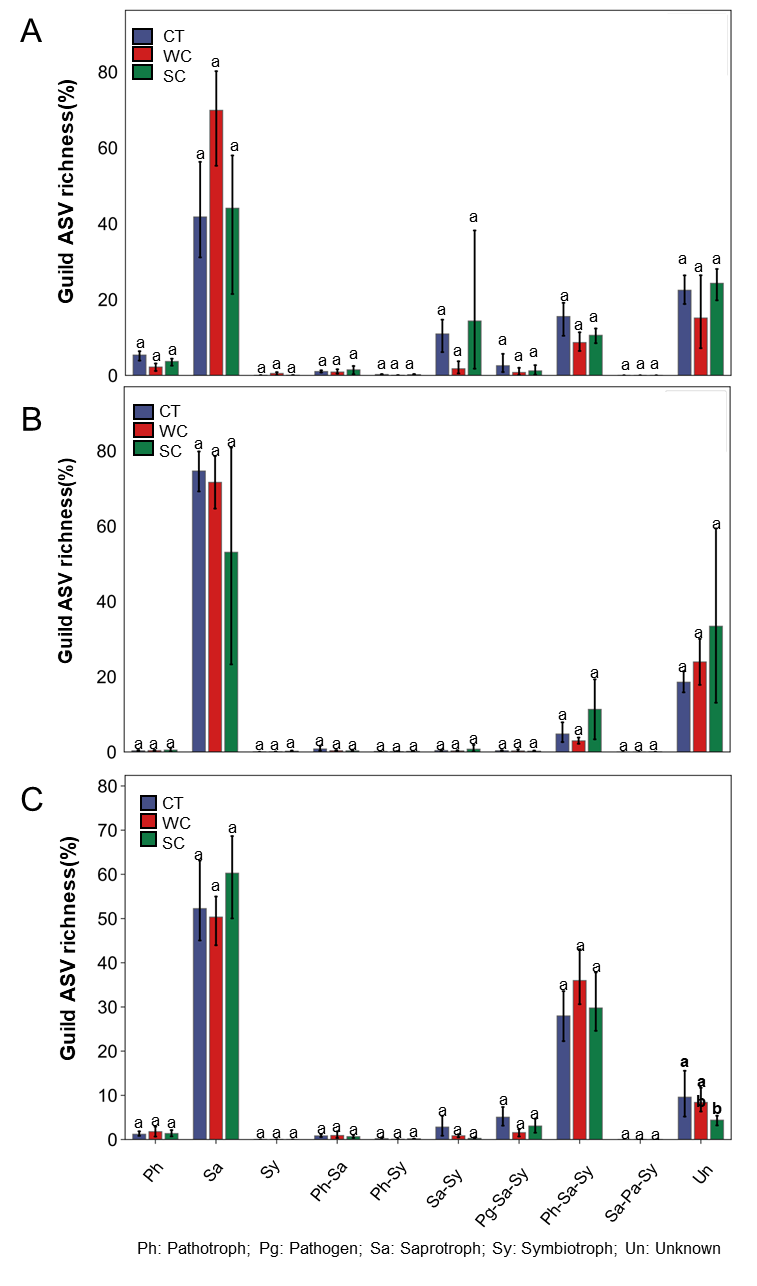


Fig. S3 Funguild predictions of functional fungal guild ASV richness of the conventional tillage (CT), weed cover (WC) and Siratro cover (SC) treatments in the furrow in 2018 (A), 2019 (B) and 2020 (C). Means within the same year followed by the same letters are not significantly different at P = 0.05 according to a protected LSD test.

Table S1 Sample DNA quantity, DNA quality and number of analyzed sequences (i.e., having sufficient quality for sequence analysis).

| Year | Area | Treatments  code | Replication | SRA accession | UV concentration  (ng/μl) | OD260/280 | OD260/230 | Analyzed sequences |
| --- | --- | --- | --- | --- | --- | --- | --- | --- |
| 2017 |  | BF | 1 | SRR22044417 | 10.40 | 1.98 | 0.37 | 39772 |
| 2017 |  | BF | 2 | SRR22044416 | 11.30 | 1.98 | 0.41 | 47000 |
| 2017 |  | BF | 3 | SRR22044405 | 13.80 | 1.37 | 0.27 | 42411 |
| 2017 |  | BF | 4 | SRR22044394 | 11.30 | 1.94 | 0.39 | 36961 |
| 2018 | Furrow | CT | 1 | SRR22044383 | 10.10 | 1.82 | 0.31 | 43220 |
| 2018 | Furrow | CT | 2 | SRR22044372 | 12.40 | 1.84 | 0.37 | 42681 |
| 2018 | Furrow | CT | 3 | SRR22044361 | 14.40 | 1.84 | 0.39 | 41060 |
| 2018 | Furrow | WC | 1 | SRR22044351 | 9.20 | 1.92 | 0.34 | 46840 |
| 2018 | Furrow | WC | 2 | SRR22044350 | 13.00 | 1.91 | 0.45 | 38380 |
| 2018 | Furrow | WC | 3 | SRR22044349 | 6.20 | 1.87 | 0.24 | 41115 |
| 2018 | Furrow | SC | 1 | SRR22044415 | 8.70 | 1.97 | 0.35 | 38536 |
| 2018 | Furrow | SC | 2 | SRR22044414 | 13.70 | 1.72 | 0.44 | 35375 |
| 2018 | Furrow | SC | 3 | SRR22044413 | 6.60 | 2.01 | 0.27 | 38077 |
| 2018 | Intercropping | CT | 1 | SRR22044412 | 9.70 | 2.06 | 0.37 | 48958 |
| 2018 | Intercropping | CT | 2 | SRR22044411 | 9.70 | 1.93 | 0.37 | 49026 |
| 2018 | Intercropping | CT | 3 | SRR22044410 | 9.80 | 1.84 | 0.34 | 45020 |
| 2018 | Intercropping | WC | 1 | SRR22044409 | 7.20 | 1.98 | 0.24 | 38658 |
| 2018 | Intercropping | WC | 2 | SRR22044408 | 11.30 | 1.91 | 0.38 | 39662 |
| 2018 | Intercropping | WC | 3 | SRR22044407 | 12.20 | 1.79 | 0.40 | 40378 |
| 2018 | Intercropping | SC | 1 | SRR22044406 | 8.60 | 1.90 | 0.30 | 40766 |
| 2018 | Intercropping | SC | 2 | SRR22044404 | 7.20 | 1.94 | 0.28 | 37483 |
| 2018 | Intercropping | SC | 3 | SRR22044403 | 8.10 | 1.96 | 0.32 | 39216 |
| 2019 | Furrow | CT | 1 | SRR22044402 | 8.37 | 1.96 | 0.23 | 30662 |
| 2019 | Furrow | CT | 2 | SRR22044401 | 9.30 | 1.84 | 0.26 | 28871 |
| 2019 | Furrow | CT | 3 | SRR22044400 | 8.30 | 1.89 | 0.24 | 33864 |
| 2019 | Furrow | WC | 1 | SRR22044399 | 9.77 | 1.87 | 0.27 | 36514 |
| 2019 | Furrow | WC | 2 | SRR22044398 | 9.73 | 1.93 | 0.29 | 33463 |
| 2019 | Furrow | WC | 3 | SRR22044397 | 8.90 | 1.94 | 0.27 | 34300 |
| 2019 | Furrow | WC | 4 | SRR22044396 | 9.73 | 1.96 | 0.28 | 34039 |
| 2019 | Furrow | SC | 1 | SRR22044395 | 9.13 | 1.99 | 0.24 | 42831 |
| 2019 | Furrow | SC | 2 | SRR22044393 | 11.40 | 1.87 | 0.30 | 38989 |
| 2019 | Furrow | SC | 3 | SRR22044392 | 10.83 | 1.86 | 0.29 | 31283 |
| 2019 | Furrow | SC | 4 | SRR22044391 | 10.40 | 1.89 | 0.28 | 40442 |
| 2019 | Intercropping | CT | 1 | SRR22044390 | 7.77 | 1.97 | 0.21 | 34675 |
| 2019 | Intercropping | CT | 2 | SRR22044389 | 11.87 | 1.95 | 0.31 | 66130 |
| 2019 | Intercropping | CT | 3 | SRR22044388 | 10.10 | 1.86 | 0.27 | 41689 |
| 2019 | Intercropping | CT | 4 | SRR22044387 | 10.60 | 2.00 | 0.29 | 34834 |
| 2019 | Intercropping | WC | 1 | SRR22044386 | 8.53 | 1.94 | 0.22 | 32331 |
| 2019 | Intercropping | WC | 2 | SRR22044385 | 9.97 | 1.88 | 0.25 | 34696 |
| 2019 | Intercropping | WC | 3 | SRR22044384 | 9.43 | 1.96 | 0.24 | 36285 |
| 2019 | Intercropping | WC | 4 | SRR22044382 | 10.40 | 1.94 | 0.27 | 37131 |
| 2019 | Intercropping | SC | 1 | SRR22044381 | 11.37 | 1.91 | 0.29 | 40588 |
| 2019 | Intercropping | SC | 2 | SRR22044380 | 9.80 | 1.92 | 0.25 | 32050 |
| 2019 | Intercropping | SC | 3 | SRR22044379 | 14.00 | 1.93 | 0.34 | 35161 |
| 2019 | Intercropping | SC | 4 | SRR22044378 | 13.00 | 1.91 | 0.34 | 32612 |
| 2020 | Furrow | CT | 1 | SRR22044377 | 3.73 | 2.13 | 0.09 | 41617 |
| 2020 | Furrow | CT | 2 | SRR22044376 | 5.63 | 2.12 | 0.14 | 82393 |
| 2020 | Furrow | CT | 3 | SRR22044375 | 5.50 | 2.08 | 0.14 | 61989 |
| 2020 | Furrow | CT | 4 | SRR22044374 | 5.90 | 2.04 | 0.15 | 58094 |
| 2020 | Furrow | WC | 1 | SRR22044373 | 5.90 | 2.01 | 0.13 | 50690 |
| 2020 | Furrow | WC | 2 | SRR22044371 | 6.87 | 2.11 | 0.17 | 57231 |
| 2020 | Furrow | WC | 3 | SRR22044370 | 4.57 | 2.05 | 0.11 | 55138 |
| 2020 | Furrow | WC | 4 | SRR22044369 | 6.50 | 1.90 | 0.16 | 53660 |
| 2020 | Furrow | SC | 1 | SRR22044368 | 8.07 | 1.91 | 0.18 | 55488 |
| 2020 | Furrow | SC | 2 | SRR22044367 | 8.50 | 2.02 | 0.20 | 62173 |
| 2020 | Furrow | SC | 3 | SRR22044366 | 11.50 | 1.86 | 0.26 | 51395 |
| 2020 | Furrow | SC | 4 | SRR22044365 | 10.30 | 1.85 | 0.23 | 44925 |
| 2020 | Intercropping | CT | 1 | SRR22044364 | 2.63 | 2.06 | 0.06 | 47311 |
| 2020 | Intercropping | CT | 2 | SRR22044363 | 3.80 | 2.09 | 0.09 | 46421 |
| 2020 | Intercropping | CT | 3 | SRR22044362 | 5.67 | 2.09 | 0.13 | 53275 |
| 2020 | Intercropping | CT | 4 | SRR22044360 | 3.30 | 2.04 | 0.08 | 56862 |
| 2020 | Intercropping | WC | 1 | SRR22044359 | 4.60 | 2.06 | 0.11 | 50841 |
| 2020 | Intercropping | WC | 2 | SRR22044358 | 3.80 | 1.88 | 0.09 | 45971 |
| 2020 | Intercropping | WC | 3 | SRR22044357 | 3.57 | 2.06 | 0.09 | 59204 |
| 2020 | Intercropping | WC | 4 | SRR22044356 | 4.97 | 2.07 | 0.13 | 57517 |
| 2020 | Intercropping | SC | 1 | SRR22044355 | 6.90 | 1.87 | 0.16 | 53291 |
| 2020 | Intercropping | SC | 2 | SRR22044354 | 7.80 | 1.89 | 0.18 | 51611 |
| 2020 | Intercropping | SC | 3 | SRR22044353 | 7.03 | 1.97 | 0.17 | 50552 |
| 2020 | Intercropping | SC | 4 | SRR22044352 | 10.20 | 1.87 | 0.24 | 54654 |

Table S2 The quality of the sequence of each sample

| Year | Area | Treatments  code | Replication | Quality filtered reads | NGS reads | SE/PE reads |
| --- | --- | --- | --- | --- | --- | --- |
| 2017 |  | BF | 1 | 40025 | 86938 | 21344370 |
| 2017 |  | BF | 2 | 47706 | 106068 | 25880699 |
| 2017 |  | BF | 3 | 42997 | 98998 | 23995373 |
| 2017 |  | BF | 4 | 37970 | 88252 | 21404684 |
| 2018 | Furrow | CT | 1 | 43926 | 65910 | 18825235 |
| 2018 | Furrow | CT | 2 | 43244 | 62862 | 18140532 |
| 2018 | Furrow | CT | 3 | 41428 | 75292 | 21669775 |
| 2018 | Furrow | WC | 1 | 48992 | 81594 | 22905950 |
| 2018 | Furrow | WC | 2 | 39344 | 72898 | 20532167 |
| 2018 | Furrow | WC | 3 | 42719 | 72744 | 21061828 |
| 2018 | Furrow | SC | 1 | 39341 | 76580 | 21904970 |
| 2018 | Furrow | SC | 2 | 36672 | 89668 | 26233954 |
| 2018 | Furrow | SC | 3 | 38463 | 81776 | 23144533 |
| 2018 | Intercropping | CT | 1 | 49506 | 65964 | 18738744 |
| 2018 | Intercropping | CT | 2 | 49794 | 87904 | 24390061 |
| 2018 | Intercropping | CT | 3 | 45660 | 75604 | 20854553 |
| 2018 | Intercropping | WC | 1 | 39077 | 144880 | 40787480 |
| 2018 | Intercropping | WC | 2 | 40713 | 88776 | 23554406 |
| 2018 | Intercropping | WC | 3 | 41734 | 75490 | 20639769 |
| 2018 | Intercropping | SC | 1 | 41346 | 69910 | 19106439 |
| 2018 | Intercropping | SC | 2 | 38086 | 74294 | 20894530 |
| 2018 | Intercropping | SC | 3 | 39904 | 77302 | 21340802 |
| 2019 | Furrow | CT | 1 | 30828 | 82270 | 23101173 |
| 2019 | Furrow | CT | 2 | 29070 | 87906 | 23961701 |
| 2019 | Furrow | CT | 3 | 34177 | 70030 | 19293891 |
| 2019 | Furrow | WC | 1 | 37957 | 78476 | 21503504 |
| 2019 | Furrow | WC | 2 | 33672 | 71350 | 19752038 |
| 2019 | Furrow | WC | 3 | 34465 | 98512 | 24189842 |
| 2019 | Furrow | WC | 4 | 35042 | 95166 | 23338040 |
| 2019 | Furrow | SC | 1 | 42945 | 90650 | 22259663 |
| 2019 | Furrow | SC | 2 | 39175 | 107990 | 26518577 |
| 2019 | Furrow | SC | 3 | 31404 | 87512 | 21530493 |
| 2019 | Furrow | SC | 4 | 41278 | 94448 | 23249398 |
| 2019 | Intercropping | CT | 1 | 35168 | 88358 | 21645958 |
| 2019 | Intercropping | CT | 2 | 67406 | 78904 | 19387326 |
| 2019 | Intercropping | CT | 3 | 42497 | 83862 | 20602935 |
| 2019 | Intercropping | CT | 4 | 35280 | 106590 | 26192404 |
| 2019 | Intercropping | WC | 1 | 32734 | 108930 | 26449627 |
| 2019 | Intercropping | WC | 2 | 35169 | 105252 | 25351619 |
| 2019 | Intercropping | WC | 3 | 37069 | 94660 | 22661114 |
| 2019 | Intercropping | WC | 4 | 39353 | 88652 | 21768012 |
| 2019 | Intercropping | SC | 1 | 41122 | 91764 | 22434625 |
| 2019 | Intercropping | SC | 2 | 32686 | 92564 | 22562991 |
| 2019 | Intercropping | SC | 3 | 35952 | 82922 | 20410514 |
| 2019 | Intercropping | SC | 4 | 33389 | 87356 | 21453100 |
| 2020 | Furrow | CT | 1 | 55008 | 116260 | 31253829 |
| 2020 | Furrow | CT | 2 | 50705 | 110466 | 29540376 |
| 2020 | Furrow | CT | 3 | 66644 | 139914 | 36329710 |
| 2020 | Furrow | CT | 4 | 63080 | 134878 | 35317591 |
| 2020 | Furrow | WC | 1 | 42234 | 89482 | 24037858 |
| 2020 | Furrow | WC | 2 | 86677 | 185738 | 47814070 |
| 2020 | Furrow | WC | 3 | 65951 | 138936 | 36084303 |
| 2020 | Furrow | WC | 4 | 58404 | 122348 | 32752412 |
| 2020 | Furrow | SC | 1 | 53163 | 112262 | 30051491 |
| 2020 | Furrow | SC | 2 | 58945 | 122968 | 32253595 |
| 2020 | Furrow | SC | 3 | 58700 | 122008 | 32121723 |
| 2020 | Furrow | SC | 4 | 57033 | 119810 | 32433508 |
| 2020 | Intercropping | CT | 1 | 51772 | 110450 | 29382580 |
| 2020 | Intercropping | CT | 2 | 50376 | 109576 | 28313798 |
| 2020 | Intercropping | CT | 3 | 57044 | 121926 | 31085964 |
| 2020 | Intercropping | CT | 4 | 59125 | 126348 | 33273302 |
| 2020 | Intercropping | WC | 1 | 57263 | 122234 | 33573149 |
| 2020 | Intercropping | WC | 2 | 57161 | 121124 | 32430953 |
| 2020 | Intercropping | WC | 3 | 53206 | 112732 | 30612800 |
| 2020 | Intercropping | WC | 4 | 58142 | 124650 | 33782422 |
| 2020 | Intercropping | SC | 1 | 58155 | 122266 | 32589642 |
| 2020 | Intercropping | SC | 2 | 66468 | 142246 | 36371465 |
| 2020 | Intercropping | SC | 3 | 53664 | 136618 | 36924667 |
| 2020 | Intercropping | SC | 4 | 47410 | 147246 | 38242074 |

Table S3 The 30 most abundant fungal genera with conventional tillage (CT), weed cover (WC) and Siratro cover (SC) treatments in the intercropping area in 2020.

| Genus | CT | WC | SC |
| --- | --- | --- | --- |
| *Fusarium* | 18.63±4.62a | 33.6±7.64a | 20.16±1.75a |
| *Mortierella* | 0.99±0.47b | 1.5±0.46b | 18.44±11.75a |
| *Humicola* | 3.54±1.39a | 6.27±1.77a | 4.28±1.23a |
| *Fusicolla* | 0.43±0.11b | 12.3±5.2a | 0.58±0.11b |
| *Acremonium* | 2.03±1.94ab | 0.11±0.05b | 5.42±1.69a |
| *Alternaria* | 4.75±2.14a | 0.41±0.13a | 1.45±1.06a |
| *Penicillium* | 1.54±0.78a | 1.57±0.23a | 1.58±1.14a |
| *Myrothecium* | 2.9±2.06a | 0.74±0.66a | 0±0b |
| *Exserohilum* | 2.58±1.54a | 0.52±0.25ab | 0.01±0.01b |
| *Aspergillus* | 0.76±0.44a | 0.69±0.28a | 1.22±0.81a |
| *Chaetomium* | 0.41±0.19a | 0.64±0.4a | 1.57±0.76a |
| *Plectosphaerella* | 0.11±0.11b | 0.18±0.12b | 2.18±0.67a |
| *Trechispora* | 1.89±1.11a | 0±0b | 0.49±0.43ab |
| *Naganishia* | 0.4±0.19a | 0.96±0.71a | 0.48±0.36a |
| *Neurospora* | 0.41±0.39a | 1.1±1.03a | 0.1±0.04a |
| *Mycosphaerella* | 0.79±0.17a | 0.21±0.11b | 0.61±0.24ab |
| *Metarhizium* | 0±0b | 1.09±0.82a | 0.49±0.4a |
| *Micropsalliota* | 0.08±0.04b | 1.06±0.45a | 0.04±0.02b |
| *Nigrospora* | 0.19±0.05b | 0.71±0.12a | 0.25±0.08b |
| *Staphylotrichum* | 0.12±0.12a | 0.68±0.37a | 0.24±0.14a |
| *Acrocalymma* | 0.49±0.27ab | 0.06±0.02b | 0.49±0.12a |
| *Pyrenochaetopsis* | 0.42±0.28ab | 0.57±0.33a | 0.03±0.01b |
| *Corynascella* | 0.15±0.05ab | 0.74±0.42a | 0.01±0.01b |
| *Poaceascoma* | 0.15±0.12ab | 0.68±0.25a | 0.03±0.02b |
| *Dokmaia* | 0.06±0.04b | 0.46±0.13a | 0.3±0.11ab |
| *Sagenomella* | 0.41±0.24a | 0.23±0.06a | 0.16±0.09a |
| *Trichoderma* | 0.29±0.2a | 0.21±0.1a | 0.21±0.07a |
| *Pseudallescheria* | 0.22±0.12a | 0.36±0.32a | 0.03±0.03a |
| *Saitozyma* | 0.19±0.15a | 0.29±0.25ab | 0.01±0b |
| *Lasiodiplodia* | 0.19±0.11a | 0.15±0.1a | 0.11±0.05a |

Same letters in the rows indicates no significant difference at P = 0.05 between treatments according to the Kruskal-Wallis rank sum test.

Table S4 The 30 most abundant fungal genera with conventional tillage (CT), weed cover (WC) and Siratro cover (SC) treatments in the furrow in 2020.

| Genus | CT | WC | SC |
| --- | --- | --- | --- |
| *Fusarium* | 21.44±4.16a | 29.62±5.17a | 24.29±2.45a |
| *Trechispora* | 1.5±0.74b | 3.14±1.81b | 25.57±7.93a |
| *Penicillium* | 8.66±6.9a | 3.03±1.01a | 11.12±5.06a |
| *Acremonium* | 5.09±1.26a | 1.6±0.5a | 3.1±0.99a |
| *Acrocalymma* | 4.6±3.65a | 0.62±0.23ab | 0.29±0.05b |
| *Trichoderma* | 0.92±0.21a | 2.67±1.87a | 1.53±0.51a |
| *Micropsalliota* | 0.54±0.29b | 4.18±3.7a | 0.03±0.03ab |
| *Humicola* | 0.82±0.16a | 1.73±0.8a | 0.89±0.2a |
| *Mortierella* | 2.37±1.38a | 0.77±0.25a | 0.17±0.1a |
| *Alternaria* | 0.73±0.48a | 0.37±0.16a | 1.08±0.56a |
| *Chrysosporium* | 0.24±0.17a | 1.16±0.96a | 0.33±0.27a |
| *Pyrenochaetopsis* | 0.79±0.25a | 0.35±0.04a | 0.44±0.18a |
| *Cladorrhinum* | 0.07±0.06a | 0.02±0.02a | 1.44±1.44a |
| *Chaetomium* | 0.13±0.04a | 0.88±0.29a | 0.14±0.03a |
| *Aspergillus* | 0.54±0.17a | 0.44±0.21a | 0.15±0.02a |
| *Mycosphaerella* | 0.32±0.15a | 0.16±0.06a | 0.61±0.21a |
| *Fusicolla* | 0.19±0.08b | 0.56±0.08a | 0.07±0.02b |
| *Lasiodiplodia* | 0.06±0.03a | 0.71±0.69a | 0.01±0a |
| *Nigrospora* | 0.36±0.18a | 0.21±0.1ab | 0.06±0.02b |
| *Purpureocillium* | 0.13±0.11a | 0.1±0.03a | 0.4±0.31a |
| *Sagenomella* | 0.19±0.08a | 0.22±0.04a | 0.2±0.13a |
| *Plectosphaerella* | 0.3±0.1a | 0.08±0.04a | 0.17±0.08a |
| *Naganishia* | 0.11±0.06a | 0.24±0.09a | 0.15±0.12a |
| *Arthrographis* | 0.01±0.01a | 0.01±0a | 0.46±0.43a |
| *Metarhizium* | 0.03±0.03ab | 0.39±0.29a | 0±0.01b |
| *Exidia* | 0.24±0.24a | 0.03±0.03a | 0.05±0.03a |
| *Dokmaia* | 0.14±0.08a | 0.07±0.03a | 0.08±0.04a |
| *Papiliotrema* | 0.11±0.05a | 0.02±0.01a | 0.1±0.03a |
| *Idriella* | 0.09±0.04a | 0.06±0.03a | 0.07±0.03a |
| *Podospora* | 0.16±0.07a | 0.03±0.02ab | 0.01±0.01b |

Same letters in the rows indicates no significant difference at P = 0.05 between treatments according to the Kruskal-Wallis rank sum test.

Table S5 PERMANOVA of the conventional tillage (CT), weed cover (WC) and Siratro cover (SC) treatments in the intercropping area and furrow in 2018, 2019 and 2020 to determine suitability for co-occurrence network analysis.

| Year | Location | Comparison | F.Model | R^2^ | Pr (>F) |
| --- | --- | --- | --- | --- | --- |
| 2018 | Intercropping area | CT-WC-SC | 1.14 | 0.28 | 0.25 |
|  |  | CT-WC | 0.69 | 0.15 | 0.90 |
|  |  | CT-SC | 1.60 | 0.29 | 0.10 |
|  |  | WC-SC | 1.22 | 0.23 | 0.20 |
|  | Furrow | CT-WC-SC | 1.27 | 0.30 | 0.13 |
|  |  | CT-WC | 1.60 | 0.29 | 0.20 |
|  |  | CT-SC | 1.16 | 0.22 | 0.40 |
|  |  | WC-SC | 1.08 | 0.21 | 0.40 |
| 2019 | Intercropping area | CT-WC-SC | 1.41 | 0.24 | 0.001 |
|  |  | CT-WC | 1.12 | 0.16 | 0.11 |
|  |  | CT-SC | 1.47 | 0.20 | 0.03 |
|  |  | WC-SC | 1.66 | 0.22 | 0.03 |
|  | Furrow | CT-WC-SC | 0.91 | 0.18 | 0.52 |
|  |  | CT-WC | 0.68 | 0.12 | 0.69 |
|  |  | CT-SC | 1.00 | 0.17 | 0.39 |
|  |  | WC-SC | 1.00 | 0.14 | 0.51 |
| 2020 | Intercropping area | CT-WC-SC | 2.56 | 0.36 | 0.001 |
|  |  | CT-WC | 2.94 | 0.33 | 0.03 |
|  |  | CT-SC | 1.97 | 0.25 | 0.04 |
|  |  | WC-SC | 2.80 | 0.32 | 0.03 |
|  | Furrow | CT-WC-SC | 2.32 | 0.34 | 0.004 |
|  |  | CT-WC | 1.05 | 0.15 | 0.41 |
|  |  | CT-SC | 2.73 | 0.31 | 0.03 |
|  |  | WC-SC | 3.00 | 0.31 | 0.03 |
